# Validation of the Medication Adherence Rating Scale in homeless patients with schizophrenia: Results from the French Housing First experience

**DOI:** 10.1038/srep31598

**Published:** 2016-08-18

**Authors:** K. Zemmour, A. Tinland, M. Boucekine, V. Girard, S. Loubière, N. Resseguier, G. Fond, P. Auquier, L. Boyer, French Housing First Study Group, T. Apostolidis, P. Birmes, T. Bossetti, R. Bouloudnine, B. Combes, J. Debieve, B. Falissard, T. Greacen, C. Laval, C. Lancon, P. Le Cardinal, J. Mantovani, D. Moreau, J. Naudin, P. Rhunter, B. Videau

**Affiliations:** 1 Aix-Marseille Univ., EA 3279 Research Unit, 13385 Marseille, France; 2 Department of Psychiatry, Sainte-Marguerite University Hospital, Marseille, France; 3 Université Paris-Est Créteil, Pôle de psychiatrie des hôpitaux universitaires H Mondor, INSERM U955, Eq Psychiatrie Génétique, Fondation FondaMental Fondation de coopération scientifique en santé mentale, Pole de Psychiatrie, Hôpital A. Chenevier, 40 rue de Mesly, Créteil, F-94010, France; 4 Department of Public Health, University Hospital, Marseille, France; 5 Aix-Marseille University, LPS EA 849, 13621, Aix-en-Provence, France; 6 Toulouse University, Toulouse, France; 7 CHU Toulouse, Toulouse,, France; 8 Etablissement Public de Santé Mentale Lille-Métropole, Lille, France; 9 INSERM U669, Maison de Solenn, University Paris-Sud and University Paris-Descartes, Paris, France; 10 Laboratoire de Recherche, Etablissement Public de Santé Maison Blanche, Paris, France; 11 CHS de la Savoie, Chambery, France; 12 École des Hautes Études en Sciences Sociales, Laboratoire interdisciplinaire d’études sur les réflexivités, Paris, France

## Abstract

The Medication Adherence Rating Scale (MARS) is one of the most widely used measurements of adherence in schizophrenia (SZ), but there is no available data regarding its psychometric properties in homeless SZ patients (HSZ). The aim of this study was therefore to assess the psychometric properties of the MARS in a large multicenter sample of HSZ subjects. This multi-centre prospective study was conducted in the following 4 French cities: Lille, Marseille, Paris and Toulouse. Three hundred and fifty-three patients were included. The 3-factor structure of the MARS was confirmed using confirmatory factor analysis: RMSEA = 0.045, CFI = 0.98, TLI = 0.97 and WRMR = 0.76. The unidimensionality of each factor was supported by the satisfactory INFIT statistics. Item internal consistencies were all higher than 0.20 and the Kuder–Richardson were higher than to 0.6, except for factor 2, which was closed to 0.5. Significant associations with symptoms, functioning and quality of life showed satisfactory external validity. The acceptability was satisfactory with missing data lower than 5% for each dimension. The MARS is a short self-administered instrument with acceptable psychometric properties in homeless SZ patients that yields interesting information about medication adherence.

Medication non-adherence has often been estimated at barely greater than 50% in patients with schizophrenia (SZ)^1^,^2^,^3^, leading to higher rates of relapse and hospitalization as well as to decreasing cognitive and functional prognosis^4^,^5^,^6^. Medication non-adherence is particularly pregnant in SZ marginalized populations, such as homeless individuals, who may face substantial barriers to medication adherence^7^. The definition of homelessness varies and may include individuals who lack a fixed, regular, and adequate nighttime residence^8^. Folsom and Jeste summarized studies about schizophrenia in the homeless from 1966 to 2001 and found a prevalence range of schizophrenia from 1% to 45%^8^. SZ has been shown to be the psychiatric disorder with the highest risk for homelessness^9^. The assessment of medication non-adherence in homeless SZ (HSZ) patients is thus an important challenge for clinicians, especially to propose targeted interventions to improve it. HSZ subjects may differ from SZ subjects as they have more comorbid substance use disorders that may impact adherence into treatment^10^. They also have different family support^11^. Family support has been shown to be crucial for adherence intro treatment in SZ subjects^12^. HSZ subjects have more emergency department use, which suggests more severe symptomatology in this population^13^. Additional research is needed on effective and practical approaches to improving health outcomes for homeless people with serious mental illness^14^. Evaluating adherence into treatment in HSZ is therefore a urgent need and has been rarely explored in the past^15^,^16^.

Three strategies have been suggested to reliably assess adherence in non-homeless SZ patients, of which none can be considered to date as a “gold standard”^17^,^18^. First, direct measure like electronic pillbox monitoring devices provides an objective measure of medication manipulation by the patient, but this strategy cannot confirm drug intake. Moreover it is expensive and therefore its use is limited in current practice. Second, plasma levels are more direct measures of medication absorption but assessing plasma levels remains costly and its interest in current practice is limited by pharmacokinetic discrepancies between subjects and by a phenomenon known as “white-coat adherence”, in which patients improve their medication-taking behavior in the 5 days before and after an appointment with the health care provider^19^. The last method based on self-report questionnaires are generally considered as the most cost-effective and time-efficient way to assess medication adherence, although it has also been reported to sometimes overestimate adherence^17^. This last option appears as the quickest and the most cost-effective mean to assess medication non-adherence in HSZ subjects.

The Medication Adherence Rating Scale (MARS) is one of the most widely used measurements of adherence in SZ patients^20^. This is a 10-item self-reported questionnaire resulting from the combination of the Medication Adherence Questionnaire^21^ and the Drug Attitude Inventory^22^, that were validated in patients with psychosis. Although initial results suggested that the scale had good reliability and validity, its psychometric properties were examined in a sample of moderate size (n = 66) and included patients with both bipolar disorder and SZ. More recently, Fialko *et al.*^23^, explored the MARS psychometric properties in a larger sample of 277 patients with SZ, schizoaffective disorder or delusional disorder. They replicated the three factors reported by previous research but also found the internal consistency of the MARS to be lower than original observations.

The aim of this study was therefore to investigate (i) construct validity and reliability, (ii) external validity and (iii) acceptability of the MARS in a large multicenter sample of HSZ subjects.

## Methodology

### Recruitment and population

The French Housing First program is a multi-centre prospective study was conducted in the following 4 French large cities: Lille, Marseille, Paris and Toulouse^24^. The inclusion criteria were as follows: age over 18 years; absolutely homeless (i.e., no fixed place to stay for at least the past 7 nights with little likelihood of finding a place in the upcoming month) or precariously housed (housed in single room occupancy, rooming house, or hotel/motel as a primary residence AND in the past year have a history of 2 or more episodes of being Absolutely Homeless OR one episode of being absolutely homeless of at least 4 weeks duration in the past year); diagnosis of SZ by a psychiatrist based on the Diagnostic and Statistical Manual of Mental Disorders, 4th ed. (DSM-IV-TR) criteria^25^; and the ability to speak French. The exclusion criterion was a reduced capacity to consent^26^. Mobile mental health outreach teams recruited patients over a 12-month period^27^. Evaluations were performed during face-to-face interviews by psychiatrists and research assistant in the offices of the mobile mental health outreach teams, which were located in the downtown area of each city. The patient completed the MARS questionnaire and other self-reported questionnaires independently or asked for assistance to complete all or part of the questionnaires. Only data at baseline (t0) were used in the MARS validation study.

### Data collection

1. Medication adherence was assessed using the French translation of the MARS^28^. This translation followed an internationally accepted methodology into 3 steps: forward translation, backward translation, and patients’ cognitive debriefing^29^. The MARS contains 10 yes/no item and the sum of items yields a final score ranking from 0 (poor adherence to treatment) to 10 (good adherence to treatment).
2. Socio-demographic information: gender, age, marital status and academic level.
3. Clinical characteristics: Mental Health was assessed using the Modified Colorado Symptom Index (MCSI) which has been validated in homeless individuals^30^. The MCSI contains 14 items that ask about how often in the past month an individual has experienced a variety of mental health symptoms, including loneliness, depression, anxiety, and paranoid delusion. An index score for this scale is calculated by summing each response. Higher scores indicate a higher likelihood of mental health problems. Functioning was assessed using the Multnomah Community Ability Scale (MCAS) which scores 17 axes of functionality independently, rated on 5-point Likert scale^31^,^32^. In this study, we used 2 axes: 8 – Acceptance of Illness (How well does the client accept (as opposed to deny) his/her psychiatric disability?) and 14 – Medication Compliance (How frequently does the client comply with his/her prescribed medication regimen?).
4. QoL was assessed using the SQoL18 which is a self-administered questionnaire developed and validated for the specific assessment of quality of life in patients with SCZ^33^,^34^,^35^. The S-QoL has 18 items and has been validated in homeless individuals with SCZ^36^ and in several languages^34^,^37^,^38^. Scores range from 0, indicating the lowest QoL, to 100, indicating the highest QoL.
5. Drug information: antipsychotic medication (first generation antipsychotic - FGA vs. second generation antipsychotic – SGA) and the number of psychotic treatments were reported.

### Statistical analyses

Statistical analyses were performed to explore construct validity, reliability and external validity of the MARS. Descriptive statistics of the sample included frequencies and percentages of categorical variables and the means and standard deviations of continuous variables.

The construct validity of the MARS was explored using confirmatory factor analysis. The following indicators were required: the Root Mean Square Error of Approximation (RMSEA) is acceptable if <0.08 and satisfactory if <0.05, the Comparative Fit Index (CFI) and Tucker‐Lewis Index (TLI) are higher than 0.9 and the Weighted Root Mean Square Residual (WRMR) is <1.0. The unidimensionality of each dimension was assessed using a Rasch analysis. The goodness-of-fit statistics (INFIT, ranging between 0.7 and 1.3) ensured that all items of the scale measured the same concept. Item-internal consistency (IIC) was assessed by correlating each item with its scale (corrected for overlap) using point biserial correlation (measure of association between a continuous variable and a binary variable) (correlation of 0.15 to 0.50 recommended for supporting item-internal consistency^39^,^40^,^41^. Item discriminant validity (IDV) was assessed by determining the extent to which items correlate more highly with the dimensions they are hypothesized to represent than with the other ones^42^. Floor and ceiling effects were reported assessing the homogeneous repartition of the response distribution.

For each dimension scale, reliability was assessed using Kuder–Richardson Formula-20 (an equivalent of Cronbach’s alpha coefficient for binary data) (a coefficient of at least 0.7 was expected for each scale^39^).

To explore external validity, Pearson’s correlation coefficients were used to investigate relationships between dimensions of the MARS and age, MCSI, MCAS, SQoL18 and number of treatments; dimension scores of the MARS were compared across patient groups (i.e., gender, marital status, academic level, SGA) using Mann-Whitney tests. Four hypotheses were formulated: (i) self-reported adherence (measured by the MARS score) has a weak to moderate correlation with adherence measured by the clinician (measured by the axes 14 of the MCAS) ; (ii) female gender is associated with lower medication adherence; (iii) High psychotic symptomatology (measured by the MCSI), low acceptance of illness (measured by the axe 8 of the MCAS), low QoL and high number of psychotropic treatments are associated with lower medication adherence and (iv) FGA are associated with lower adherence compared to SGA.

The acceptability of the MARS was tested using the percentage of missing values.

Data analyses were performed using the PASW 17.0.2 and MPLUS 7.2 software.

### Ethical Approval

The study was carried out in accordance with the relevant guidelines and regulations including the principles of the Declaration of Helsinki, 6th revision^43^. Informed consent was obtained from all subjects. We received written consent from all the participants of our study. The Local Ethics Committee (Comité de Protection des Personnes Sud-Méditerranée V, France: trial number 11.050) and the French Drug and Device Regulation Agency (trial number 2011-A00668-33) approved this study.

## Results

### Sample characteristics

Three hundred and fifty-three homeless SZ patients completed the MARS. The mean age of the sample was 37.8 years (standard deviation = 9.8) and 14.7% of the sample was female (Table 1).

### Construct validity and reliability

The three-factor structure of the MARS (factor 1: medication adherence behavior; factor 2: attitude to taking medication score; factor 3: negative side-effects and attitudes to psychotropic medication score) was confirmed by confirmatory factor analysis. All of the indices from the confirmatory model were satisfactory (RMSEA = 0.045, CFI = 0.98, TLI = 0.97 and WRMR = 0.76). The overall scalability was satisfactory. (Table 2) All of the items showed a good fit for the Rasch model in each factor, and none of the items had a statistical INFIT outside the range of acceptability. IIC were all higher than 0.20 and the correlation of each item with its contributive factor was higher than that with the other factors (IIC > IDV), except for factor 2. Floor effects were less than 20% for factors 1 and 2. Ceiling effects were higher than 20% for factors 1 and 3. The Kuder–Richardson coefficient were higher to 0.6, except for factor 2, which was closed to 0.5.

### External validity

All of the details are provided in Table 3. External validity can be considered as satisfactory. The MARS total score and the first 2 factors (1 and 2) were moderately correlated with adherence measured by the clinician (axe 14 of the MCAS) (Pearson’s correlation: 0.18–0.25). Significant associations with respectively psychotic symptomatology (MCSI), acceptance of illness (axe 8 of the MCAS), QoL, and with the total number of psychotropic treatments showed satisfactory discriminant validity (all p < 0.05). A statistical trend (p = 0.052) was found in favor of a better adherence with SGA than with FGA (Table 3).

### Acceptability

The acceptability was satisfactory with missing data lower than 5% for each dimension.

## Discussion

Altogether, our results may be summarized as follows: in a sample of 353 HSZ subjects, the psychometric properties of the MARS were globally satisfactory, except for some indicators that suggest that minor changes could improve its performance. The external validity confirmed the clinical interest of the MARS.

### Construct validity and reliability

The present work confirmed the 3-factor structure of the MARS and the unidimensionality of each factor supported by satisfactory INFIT statistics. This scale suggests that adherence to pharmacological treatment is complex and includes the effective behavior toward treatment intake (factor 1), believes toward medication (factor 2) and subjective negative effects of pharmacological treatments (factor 3). IIC values were satisfactory (i.e., higher than 0.2) but factor 2 had IDV values superior to the IIC values. This may be due to some items that are very close between the different factors. For example, the item 5, « I take my medication only when I am sick » included in factor 2, is closed to item 3 “When you feel better, do you sometimes stop taking your medication? » included in factor 1. Item 7 “My thoughts are clearer on medication » (also included in factor 2) is closed to items 9 and 10 « I feel weird, like a ‘zombie’ on medication » « Medication makes me feel tired and sluggish », both included in factor 3 (Table 2).

The Kuder–Richardson coefficient were higher than 0.6, similar to the values produced by Fialko *et al.*^23^ and lower to the values produced by Thompson *et al.* (2000) during the original development of the scale (global alpha = 0.75 and 0.72 in our study)^20^. However, this finding may not represent a weakness of the reliability of the scale^23^. Indeed, a low value of alpha could be due to a low number of questions and/or poor inter-relatedness between items^44^. It is thus likely that the brevity of the MARS is partly responsible for the low value of alpha. Nevertheless, coefficient of 0.6 could be acceptable^45^. The ceiling and floor effects were higher than 15% for some factors, suggesting that the MARS is probably not optimal to discriminate between subjects at either extreme of the scale. However, this finding is not surprising considering the small number of item and the binary response format.

The construct validity and reliability of the MARS could thus be improved by adding more response options (e.g., Likert scale format) or by adding more items more relevant for homeless individuals, which could also be beneficial for other important properties such as sensitivity to change^46^. However, these changes should not compromise the quick and simple format of the MARS. Future studies should explore the different options.

### External validity

We made 4 hypotheses about external validity. External validity, which was explored by using socio-demographic characteristics and psychiatric and adherence measures, confirmed the majority of our assumptions. Some discrepancies were found, but can be explained by the characteristics of HSZ vs. SZ subjects (e.g., less family support, more substance abuse, lower insight into illness) (Table 3).

First, we hypothesized that self-reported adherence (measured by the MARS score) may have a weak to moderate correlation with key-worker-rated adherence (measured by the MCAS). This difference between key-worker and self-reported adherence is consistent with Fialko *et al.* findings^23^ and suggests the need for assessing specifically self-reported adherence in HSZ patients.

Secondly, we hypothesized that female gender would be associated with poor adherence. Indeed, female is a particularly vulnerable group, and initiation of treatment and monitoring are often difficult for them^47^,^48^,^49^. Female are in the minority of the homeless population and they often enter services at a much later stage than male, and when their problems have become more severe and enduring^50^. In our study, females have lower score for the factor 3, i.e., that females are more sensitive to negative side-effects and have more negative attitudes to psychotropic medication: “I feel weird, like a zombie, on medication”, “Medication makes me feel tired and sluggish”. This result suggests that interventions in female SZ patients should ensure that treatment is effective and that side effects are correctly managed with regular follow-up. Promoting a positive therapeutic alliance and good communication between clinician and patient is probably extremely important to improve individual perceptions or beliefs about the treatments. The side effects are probably not the only problem, and the lack of knowledge about each side effect and lack of skills or management strategies to cope with side effects are probably as much important^2^,^51^,^52^.

Thirdly, we hypothesized that high psychotic symptomatology (measured by the MCSI), low acceptance of disease (measured by the MCAS), low QoL and high number of psychotropic treatments would be associated with lower medication adherence. As expected, high psychotic symptomatology, low acceptance of disease and low QoL was associated with lower medication adherence consistently with previous studies^53^,^54^,^55^. Contrary to our hypothesis, we found that the number of psychotropic treatments was positively correlated with adherence scores. It may be advanced that homeless SZ patients identified by their doctors as more adherent are prescribed more medications^23^. They may receive a more intensive management because of their better acceptance.

Lastly, we hypothesized that SGA would be associated with a better adherence compared to FGA. Our results seemed confirm this hypothesis (statistical trend). This is consistent with the findings of Dassa *et al.*^54^. This result may be explained by a better tolerability of SGA compared to FGA, which may improve the adherence into treatment. However SGA remains a heterogeneous class and further studies should determine which antipsychotics are specifically associated with better adherence compared to others.

### Acceptability

Another important finding is the excellent acceptability of the MARS. The rate of missing data was particularly low, especially for homeless patients. Several explanations may be proposed. The MARS is one of the shortest instruments among recent adherence measures for use in schizophrenia^23^. According to several authors, a short form of the scale is frequently associated with improved acceptability^35^,^56^. The average completion time is expected to be around five minutes, which will facilitate its use in research and clinical practice.

### Limits and perspectives

An important methodological issue remains in the definition of adherence in schizophrenia, as mentioned in the rationale of the present study. Validity is considered present when the measurement predicts an external criterion based on a gold standard. In the case of adherence, there is no gold standard, so the instrument is considered valid if it consistently fits other construct. Although this choice might be debatable, we undertook comparisons with measurements of key-worker rated adherence and clinical variables well known to be associated with adherence, like acceptance of illness. The clinical significance of this study should be cautiously interpreted in accordance with this choice.

Even with the large overall sample size of this multi-centre study, the sample may not have been representative of the homeless population with schizophrenia. Because our study took place in large cities, our findings may not generalize to homeless people living in smaller cities in which life conditions and needs may be different. However, our study included southern and northern cities, thus taking into consideration socio-economic, cultural and climatic differences.

The present study is a cross-sectional study and longitudinal studies are needed to determine the predictive performance of this measure, reproducibility of these results and the sensibility to change. This last property is of major interest for the follow-up of patients in clinical practice and for psycho-educational program researches.

## Conclusion

Altogether, these results suggest that the MARS is a relevant tool to quickly assess self-reported adherence, believes toward medication and subjective negative side effects of pharmacological treatment in HSZ patients in daily practice. This scale has been well accepted and opens to a discussion between the patient and the clinician about pharmacological treatment. However, future works are necessary to improve some characteristics of this scale.

## Additional Information

**How to cite this article**: Zemmour, K. *et al.* Validation of the Medication Adherence Rating Scale in homeless patients with schizophrenia. Results from the French Housing First experience. *Sci. Rep.* **6**, 31598; doi: 10.1038/srep31598 (2016).

## Figures and Tables

**Table 1:** Socio-demographic and clinical characteristics of the study sample.

	Whole sample N = 353
Socio-demographic characteristics	
Gender (female), *n* (*%*)	52 (14.7)
Age (years), *mean* (*SD*)	37.8 (9.8)
Marital status (single), *n* (*%*)	325 (92.1)
Illness characteristics	
MCSI, *mean* (*SD*)	21.6 (12.0)
MCAS “Acceptance of Illness*, mean* (*SD*)	2.0 (1.2)
Treatment	
SGA, *n* (*28%*)	102 (40.6)
Total number of treatment*, mean* (*SD*)	2.2 (1.4)
Adherence to treatment	
MARS total score*, mean* (*SD*)	5.5 (2.6)
Medication adherence behavior score*, mean* (*SD*)	2.1 (1.4)
Attitude to taking medication score*, mean* (*SD*)	2.3 (1.2)
Negative side-effects and attitudes to psychotropic medication score*, mean* (*SD*)	1.2 (0.8)
MCAS “Medication Compliance”*, mean* (*SD*)	3.1 (1.0)
Quality of life	
SQoL18 Index*, mean* (*SD*)	47.7 (17.3)

Modified Colorado Symptom Index (MCSI); Multnomah Community Ability Scale (MCAS); Second Generation Antipsychotic (SGA); Medication Adherence Rating Scale (MARS); Schizophrenia Quality of Life questionnaire (SQoL18).

**Table 2:** Internal structural validity/reliability/unidimensionality.

Dimension (number of items)	Item-Internal Consistency (min-max)		Item Discriminant Validity (min-max)		Missing values (%)	Floor (%)	Ceiling (%)	Alpha*	Infit (Min-Max)**	
Medication adherence behavior score (4 items)	0.39	0.57	0.15	0.39	1.5	17.8	24.6	0.71	0.92	1.08
Attitude to taking medication score (4 items)	0.22	0.30	0.13	0.39	2.9	7.8	18.6	0.47	0.98	1.03
Negative side-effects and attitudes to psychotropic medication score (2 items)	0.45	0.45	0.20	0.25	2.7	26.0	46.4	0.61	0.96	0.97

^*^Cronbach’s Alpha.

^**^Rash’s statistics.

**Table 3:** External validity.

	Medication adherence behaviour	Attitude to taking medication	Negative side-effects and attitudes to psychotropic medication	MARS total score
Gender				
Male	2.1 (1.4)	2.4 (1.2)	1.3 (0.8)	5.6 (2.6)
Female	1.9 (1.6)	2.1 (1.1)	0.9 (0.9)	4.9 (2.4)
p-value	0.38	0.12	**0.02**	**0.04**
Age	0.05	0.02	0.09	0.05
Marital status				
Single	2.1 (1.4)	2.3 (1.2)	1.2 (0.8)	5.4 (2.6)
Couple	2.5 (1.5)	2.6 (1.2)	1.4 (0.8)	6.3 (2.5)
p-value	0.21	0.15	0.14	0.11
Total number of treatment	0.04	0.14**	0.06	0.12*
SGA				
Yes	2.3 (1.4)	2.3 (1.1)	1.3 (0.8)	5.8 (2.3)
No	2.1 (1.4)	2.3 (1.3)	1.1 (0.9)	5.3 (2.7)
p-value	0.41	0.68	0.05	0.13
MCAS item “Medication Compliance”	0.22**	0.18**	0.10	0.25**
MCAS item “Acceptance of Illness”	0.14**	0.13*	0.06	0.18**
MCSI score	-0.24**	-0.01	-0.12*	-0.16**
SQoL-18	0.15**	0.04	0.14*	0.14*

In Bold: statistically significant.

^*^p-value < 0.05.

^**^p-value < 0.01.
